# Estimation of the Precision of a Structured Light System in Oil Paintings on Canvas

**DOI:** 10.3390/s19224966

**Published:** 2019-11-14

**Authors:** David Sánchez-Jiménez, Fernando Buchón-Moragues, José M. Bravo, Juan V. Sánchez-Pérez

**Affiliations:** 1 Departamento de Ingeniería Cartográfica, Geodesia y Fotogrametría, Universitat Politécnica de Valéncia, Camino de Vera s/n, 46022 Valencia, Spain; dasanji@doctor.upv.es (D.S.-J.); fbuchon@upvnet.upv.es (F.B.-M.); 2 Centro de Tecnologías Físicas, Acústica, Materiales y Astrofísica, División Acústica, Universitat Politécnica de Valéncia, Camino de Vera s/n, 46022 Valencia, Spain; jobrapla@fis.upv.es

**Keywords:** pictorial artworks authentication, pictorial artworks cataloging, three-dimensional modeling, non-destructive testing, close-range photogrammetry, structured light

## Abstract

The conservation and authentication of pictorial artworks is considered an important part of the preservation of cultural heritage. The use of non-destructive testing allows the obtention of accurate information about the state of pictorial artworks without direct contact between the equipment used and the sample. In particular, the use of this kind of technology is recommended in obtaining three-dimensional surface digital models, as it provides high-resolution information that constitutes a kind of fingerprint of the samples. In the case of pictorial artworks with some kind of surface relief, one of the most useful technologies is structured light (SL). In this paper, the minimum difference in height that can be distinguished with this technology was estimated, establishing experimentally both the error committed in the measurement process and the precision in the use of this technology. This study focused on the case of oil paintings on canvas and developed a low-cost system to ensure its wide use.

## 1. Introduction

Pictorial artworks are a fundamental part of cultural heritage and, therefore, a great effort must be made to ensure their durability, protecting them from damage caused by external agents such as temperature, humidity, and different kinds of pollution. To do that, the need for preventive conservation of this kind of artwork becomes a crucial factor, with continuous monitoring or follow-up of environmental temperature and humidity. Thus, their exhibition and conservation have to take place in rooms with temperature and humidity control systems. A temperature of 19–24 °C and a humidity of 45–65% are considered ideal conditions for conservation [1]. If these conditions are not met, the deterioration of the work is quicker with damage to both the substrate (curvatures and deformations) and the paint, for example, cracks, incisions, tears, raised or peeled paint, loss of pigments that come off, etc. [2].

On the other hand, these measures should be supported by adequate and accurate documentation and surveying actions in order to continuously check the conservation state of the pictorial artworks ensuring, at the same time, their authenticity. To obtain this documentation, several technologies, usually called non-destructive testing (NDT), allow the analysis of pictorial artworks avoiding direct contact between the sample and the instruments used. Some NDT technologies provide information about the inner layers of the pictorial artworks. Among them, we can mention infrared thermography [3], X-rays [4], and ultrasounds [5]. However, obtaining three-dimensional surface digital models (3DSDM) is recommended by some authors for the preventive conservation of pictorial artworks due to the fact that a precise 3DSDM of high resolution, made up of millions of points, possesses unique geometric information of its physical characteristics [6]. Therefore, this metric document can be considered as a kind of “fingerprint” of the painting [7]. The periodic evaluation of changes in the 3DSDM could help to detect deterioration of the pictorial artworks at an early stage, improving their conservation and indicating the convenience of their restoration. Some technologies used to obtain a 3DSDM of these artworks and to model their surface layer, detecting irregularities in the canvas and its support, are raking light [8], laser scanner [9], and structured light (SL) [2,10].

Structured light is a photogrammetric technology that does not make any physical contact with the object in order to measure, and it has been used in areas as diverse as surgery [11], industry [12], and aeronautics [13]. In addition, SL has been frequently used in the documentation of cultural heritage. For instance, without pretending to be exhaustive, it has been used in the area of archaeology to recreate details of excavated surfaces and associated artifacts in two sites of the Middle Paleolithic in south-western France [14]; in the scanning of sculptures such as “Minerva of Arezzo” [15]; in architecture for obtaining three-dimensional models with photo-realistic textures of specific parts in facades of historic buildings [16] as well as in wooden maquettes of ancient Nubian temples [17]. The analysis of pictorial artworks with SL can have several useful objectives such as digitization to obtain orthoimages and high-resolution three-dimensional models [1]. In this field, SL has been extensively tested and offers a high resolution for analyzing reliefs in paintings [18] or to study deformations in Leonardo da Vinci’s work “Adoration of the Magi” [19]. Some authors have proposed the use of SL in authentication tasks, such as identifying authorship or detecting falsifications, or in cataloging works, such as classifying paintings according to the relief of their surfaces, specifically in paintings on wooden panels [7]. In addition, SL is an especially simple and fast NDT data capture system in which it is not necessary to manipulate pictorial artworks (removal of the frame, displacement of its location, etc.) to obtain their 3DSDM with the increase in security for the protection of the painting that this implies.

However, despite the increased use of SL in the analysis of pictorial artworks, it is remarkable the differences among the many NDTs available for carrying out three-dimensional modelling studies and the scarcity of works on their usage guidelines and comparative data on the results obtained. On the other hand, the specifications established by the manufacturers may cause confusion among users, and it is necessary to collect data, under practical conditions of use, on the usefulness, performance, and accuracy of these technologies [6]. In this sense, some comparative studies have been carried out on the usefulness and reliability of SL in the area of pictorial artworks where the precision obtained depended on the size of the object, and the system used varied within a wide range, from sub-millimetric to sub-centimetric accuracy [1,2,20].

The precision of SL depends on the size and the material of the surface of the object [21]. This precision may differ from the technical specifications given by the manufacturer which were taken in controlled conditions and for geometrically known-sized objects. Following this line, and taking into account the need for practically estimating the real precision obtained in these kinds of objects, in this paper, we present an experimental study to evaluate the precision of 3DSDM of pictorial artworks obtained with SL. In addition, we evaluated the potential use of SL to detect falsifications and to track the deterioration of original pictorial artworks over time. Among the wide range of commercial SL systems, we focused our study on low-cost specific hardware along with free software so that the system in general can be widely used. The results obtained are valid due to the characteristics of SL for pictorial artworks with some kind of relief.

This article is organized as follows: In Section 2, we explain the theoretical basis of SL. The specific details of the experimental setup, the characteristics of the samples, and the stages of the measurement procedure are presented in Section 3. In Section 4, the results obtained are shown and discussed. Finally, the last section contains the concluding remarks where the results are summarized.

## 2. Theoretical Basis

Structured light is an NDT based on the continuous emission of several light patterns on the sample under study and which are emitted by a video projector and captured by another video or photographic camera. With this method, the precision obtained directly depends on the spatial resolution of the captured image which can reach hundredths of a millimeter or even microns [13,18].

**Figure 1:**
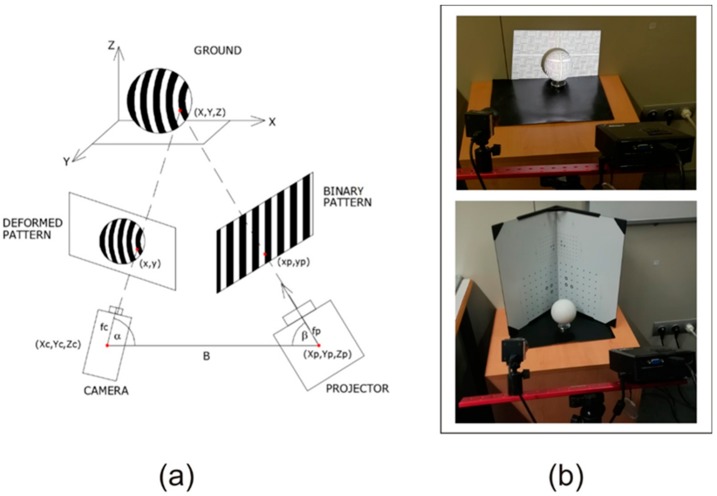
(**a**) Outline of the process for obtaining coordinates of the points in an object using structured light (SL). (**b**) Two views of the experimental setup: a detail of the deformed light pattern can be seen at the upper part, while the calibration panel is shown at the bottom.

In SL, the 3D coordinates of each point of an image are obtained by (i) capturing the light pattern distorted by the object; (ii) the knowledge of the angles between the projection and observation systems, and (iii) the length of the optical basis connecting the nodal points or projection centers of both systems. Using absolute coding technologies (such as Gray-Code), it is possible to automatically solve the problem of correspondence. Once this is known, the spatial position of each point is measured by triangulation. The geometric principles of SL are based on the projection of a series of binary light patterns on the object to be measured and the subsequent analysis of its deformation. In this way, a photogrammetric triangulation is produced between the projected image (called binary light pattern), the object, and the image captured by a camera (deformed light pattern). In Figure 1a, a scheme of the geometry of the process can be seen.

In order to measure this deformation (see the upper part of Figure 1b), it is necessary to know the geometry among the different elements involved: camera–projector distance (called baseline or B), the angles between the main directions of both the camera and the projector with the baseline, α and β, respectively, and the coordinates of both the camera (Xc, Yc, Zc) and the projector centers (Xp, Yp, Zp). All these geometrical data must be determined in a calibration process of the system on reference standards, which is carried out before the measurement. The calibration is carried out by making a set of measurements on a panel already calibrated with a known matrix of points, which is called the calibration panel (see the bottom part of Figure 1b). The principles of spatial resection and intersection are then applied in which each point on the calibration panel is defined by two lines: one defined by the projector center and the point of the image of the projected pattern and the other defined by the camera center and the point of the captured image. Considering all these parameters, a system of equations is created taking into account (i) the coordinates of the photographic or video camera center (Xc, Yc, Zc); (ii) the coordinates of the projector center (Xp, Yp, Zp); (iii) the image coordinates of the projected points (xp, yp); (iv) the image coordinates of the deformed points (xc, yc); (v) the focal distances of the projector and the camera (fp, fc); (vi) the spatial position of the projector on the coordinated trihedron (κp, φp, ωp); (vii) the spatial position of the camera on the coordinated trihedron (κc, φc, ωc); and (viii) the ground coordinates of each of the measured points (X, Y, Z).

The system of equations defines the geometrical configuration of the measuring system. These parameters are unknowns in the calibration phase and data in the measurement phase [22]. The system of equations as well as the resolution of the method can be seen in the work developed by Batlle et al. [23]. Note that once the measuring system has been calibrated and the position of the projector and camera are fixed, any change affecting the relative position among them will require a new calibration. Equation (1) shows, as an example, one of the relationships among the parameters defined above:

(1) $$\left[\begin{array}{c} X\\ Y\\ Z \end{array}\right]\;=\;\frac{B}{\mathrm{fc}\;\cot\alpha -x}\;\left[\begin{array}{c} x\\ y\\ \mathrm{fc} \end{array}\right]$$

where (X, Y, Z) are the ground coordinates of the measured point; B is the basis; α is the main direction of the camera with the baseline; fc is the focal distance of the camera, and (x, y) are the image coordinates in the camera.

## 3. Materials and Methods

### 3.1. Experimental System and Dataset

The SL scanner used in this work is the registered trademark DAVID SLS-1 (David Vision Systems GmbH, Koblenz, Germany) [24] included in the category of active scanners based on triangulation, and the type of binary light pattern projected corresponds to the time-multiplexing coding [25]. This scanner is formed by a projector ACER K11+ (ACER Inc., New Taipei City, Taiwan) which emits the binary patterns on the sample and a video camera with a resolution of 1280 × 960 pixels with a 12 mm COMPUTAR lens that records images of the different patterns projected on the sample. To reduce the noise in the measurements, DAVID SLS-1 allows the emission of a high number of binary patterns: 58 different patterns in the high-quality mode, 26 patterns in the standard mode (default option), and 22 patterns in the fast mode. The camera records each of the different projected light patterns as well as the natural image in RGB color to assign to each measured point the color code obtained in the corresponding pixel, achieving as a result a textured 3DSDM. The maximum resolution (point density) and precision (point dispersion) achievable by DAVID SLS-1 are 0.2% and 0.1% of the object size, respectively, as indicated in the manufacturer’s technical specifications [26]. A great advantage of this scanner is its high precision and acceptable working speed (approximately 45 s for each scan) at a low price which can be considered acceptable for its implementation in most cataloging, restoration, and authentication work.

Finally, the pieces of software used were DAVID-Laserscanner Pro Edition (DAVID Vision Systems GmbH, Koblenz, Germany) [24] and CloudCompare (version 2.6) [27] for obtaining and processing the 3DSDM, respectively.

In this article, we worked with the high-quality mode of the DAVID SLS-1. Therefore, the emitter projected 58 different light patterns on each sample. This means that the resulting 3DSDM for each sample was obtained from the average of 58 measurements. Since we worked with four samples and each one was measured 4 times, 16 3DSDM and 928 measurements were obtained for the elaboration of the article. Moreover, and as we will explain in the next section, we used only four paintings but painted by the same artist, which provides the worst-case scenario for detecting differences.

### 3.2. Characteristics of the Samples

**Figure 2:**
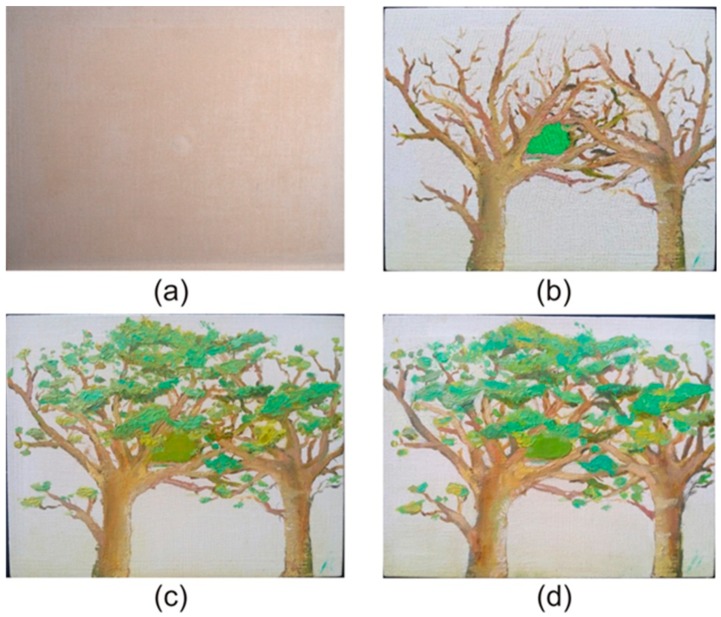
The canvases used in the study. (**a**) Initial state of the first canvas with a primer coat applied (sample A); (**b**) intermediate state of the first canvas, in which some pictorial elements were added (sample B); (**c**) completely finished painting of the first canvas (sample C); (**d**) second canvas, in which an attempt was made to imitate the painting of the first in its final stage (sample D).

The samples analyzed in this work were two canvases of the same size (230 mm wide × 175 mm high) stretched on a wooden support. The first canvas was measured in three successive stages simulating the different steps of the creation of a painting in which different layers are added. These three different steps may indicate the utility of this system to track the deterioration of the painting over time. These steps are (i) initial stage (sample A), in which a primer coat is applied; (ii) intermediate stage, in which different brushstrokes have already been applied (sample B); and (iii) final stage, which corresponds to the completely finished painting (sample C). On the other hand, and to analyze the use of DAVID SLS-1 for the authentication of paintings, a second canvas was prepared, imitating the first one in its final stage (sample D). Both canvases were painted by the same artist. The four considered samples are shown in Figure 2.

Note that, in order to control the process from the beginning, we used the paintings created by the same artist which is the best way to analyze the differences among two apparently equal paintings. Despite having used a relatively low dataset consisting of 16 3DSDMs, they were the worst-case situation, since the artist’s pictorial technique does not vary and, consequently, produces a similar 3DSDM. Obviously, we could have compared a painting created by a famous artist with a copy made by our artist, but we thought that in that case, the differences among the two copies would be greater, adding also the problem of the availability of the original painting. Thus, the comparison among the 3DSDMs of the same two paintings created by the same artist provided the least external error for our experiment.

The maximum resolution, applying the percentages given in the previous section and indicated by the manufacturer to the maximum dimensions of the canvases, could be up to 0.46 mm, while the precision could be up to 0.23 mm [26].

### 3.3. Methodology

The procedure for measuring and comparing different 3DSDMs using DAVID SLS-1 consists of the following steps:

(1) Calibrating the setup: Since DAVID SLS-1 does not have a fixed configuration, it needs to be calibrated each time it is assembled for measurement. To do that, the distance between the projector and the camera (baseline) must be previously determined according to the sample size. A first measurement should be made using the standard binary light patterns to obtain, firstly, the values of the parameters that define the photogrammetric triangulation and, secondly, the coordinates of the sample in a metric coordinate system. In this work, the values of the parameters were baseline 160 mm; distance from the scanner to the object 450 mm; and calibration template 240 mm.
(2) Obtaining the point cloud: the video projector launches a series of 58 binary light patterns with different configurations and orientations which impinge on the sample and are deformed according to its orography. Each pattern is captured by the camera as an image and, knowing the geometry of the setup, the position of each point on the scanned object is measured, first as image coordinates and then transformed into ground coordinates. Using DAVID-Laserscanner Pro Edition software, the point cloud is triangulated, generating a 3DSDM. Finally, the video projector launches three color patterns (i.e., blue, green, and red) to obtain the texture of the object and to apply it to the 3DSDM.
(3) 3DSDM noise filtering: using CloudCompare software, continuous 3DSDM data becomes discrete, selecting 1,000,000 points evenly distributed in the sample. Point cloud noise is filtered and removed.
(4) Comparing point clouds: two point clouds are registered; one of them is assigned as the reference cloud and the other as the comparison cloud. The aim of the registration is to minimize the distance between both point clouds so that they locate in the same reference system and, therefore, become comparable.

There are several methods for registering point clouds, and many of them have been improved in recent years. An initial classification of these methods could be made according to their approach: rigid or non-rigid. Rigid methods involve a rigid environment in which a transformation of 6 degrees of freedom (DOF) takes place. It consists of a displacement and a rotation of the comparison cloud with respect to the three directions of the reference space to make it match with the reference cloud. On the other hand, non-rigid methods allow for aligning objects that change their shape over time and, therefore, their transformations have more than 6 DOF. Another possible classification can be made according to the required initial conditions and with the pursued detail in the register, finding rough and fine approaches. In the first one, clouds are registered roughly regardless of their initial location, while in fine approximations a more accurate registration is carried out, requiring an initial location where both clouds are close to each other. In general, both approaches are combined to reduce the number of iterations that the fine approach needs to accurately register the comparison cloud and to increase its chances of success. In our case, we used the method of a rigid and fine approach called an iterative closest point (ICP) [28]. This algorithm works as follows: a cost function is defined which represents the current error and indicates the degree of overlap among the two clouds. After that, this cost function is iteratively minimized by estimating the combination of translation and rotation that would optimally align the clouds. The least squares method is used for this purpose in which it is possible to assign different weights at certain points and reject outliers before alignment. The translation and rotation matrix solution is obtained when several iterations are performed or when the distance among both clouds is shorter than a certain threshold.

There are several versions of the ICP algorithm, some of them including point-to-point ICP, which takes into account only the closest point of the comparison cloud to the reference cloud and is therefore more sensitive to outliers; point-to-surface ICP, which takes into account the vicinity of each point in both the comparison and the reference clouds thus increasing noise resistance; non-linear ICP, which combines the solution of least squares with the sum of absolute values; and generalized ICP (G-ICP), which is more permissive in terms of ideal data assumptions and allows greater flexibility for samples with noise. Other alternatives to register point clouds are gravitational approach (GA) [29], coherent point drift (CPD) [30], robust point matching (RPM) [31], Gaussian mixture model (GMM) [32], principal component analysis (PCA) [33], singular value decomposition (SVD) [34], and K-D tree [35].

Many comparative studies have been developed on the execution time and precision of the algorithms. Some of them have been systematic reviews or benchmark surveys [36,37] while others proposed new variants of known algorithms [38,39]. The work of Zhu et al. [36] represents an excellent review of the available methods in which several experiments with some representative point set registration algorithms are performed, obtaining the best results for the CPD-GL algorithm. In the study by Bellekens et al. [37], the accuracy and precision of 6 different rigid methods (PCA, SVS, 3 variants of ICP, and a combination of SVD + ICP point-to-point) is compared, with the best results in accuracy for SVD + ICP point-to-point, and in precision for ICP point-to-surface. Other works present a new option for GA with which they obtained better RMS than using other traditional methods of ICP, GA or CPD [38]. A new alternative to the SVD method has been developed by other researchers in which point clouds are transformed into images and a SURF algorithm is used to detect homologous pixels, obtaining similar precision results to those of G-ICP and 10 times faster [39].

As a conclusion, we chose the ICP algorithm for this study because it is one of the most used methods in many applications, including 3D modelling of paintings [2,20,40], and it is available in the CloudCompare software used in this work.

## 4. Results and Discussion

In order to estimate the precision of the 3DSDM of oil paintings on canvas obtained through SL, we carried out a two-step research process. The first step was performed under controlled conditions, obtaining all the 3DSDMs of the aforementioned samples using the same calibration conditions for each sample, and its objective was to estimate the experimental precision of the 3DSDM obtained with the low-cost equipment used, independently of the specifications that appear in its technical documentation. The second step used independent calibration conditions for each 3DSDM obtained, and its goal was to check the possibility of using SL to detect falsifications or to evaluate deteriorations of pictorial artworks. This second step gives information about the experimental repeatability of the process, since it allows us to establish the percentage of similarity between two 3DSDMs of the same pictorial artwork obtained on different dates. This percentage would allow us to validate if the 3DSDM obtained with SL can constitute a fingerprint of the pictorial artwork.

For that purpose, each one of the samples shown in Figure 2 was measured four times, obtaining 16 3DSDMs. The first three measurements were made without varying the position of the equipment with respect to the sample and were used for the first experiment. These 3DSDMs were named using the letter of the sample and the number of the measurement (e.g., for sample A, the first three measurements were named A1, A2, and A3). The fourth measurement of each sample was obtained after recalibrating the setup and was used for the second experiment. These 3DSDM measurements were named with the letter of the sample and an apostrophe (e.g., A’).

To quantify the experimental precision of the SL system, we analyzed the similarity among the measurements of a sample. To do that, we calculated the percentage of points that had the same height in each pair of 3DSDMs. This proportion is given by the cumulative relative frequency (Hi). This parameter is defined, for a dataset, as the number of scores that are equal to or less than a certain value. The Hi indicates, in the comparison of a pair of 3DSDMs, the proportion of points of one of them that are at a distance shorter than a certain value in comparison with the other 3DSDMs. When comparing two identical samples, the ideal result would be Hi = 1 (i.e., 100%) for a difference in height (Δh) of 0 mm; that would mean that the point cloud of both 3DSDMs was exactly in the same position. The precision of the SL system was estimated as the minimum Δh that consistently achieves a Hi close to 1 (neglecting the outliers of the 3DSDM that prevent Hi from being exactly 1).

In the first step, we determined the precision of the SL system under controlled conditions, i.e., sharing calibration in their acquisition. To indirectly quantify this precision, we analyzed the Δh among the point clouds of the different 3DSDM pairs of each sample (e.g., A1–A2; A1–A3; A2–A3), obtaining a total result of 12 combinations. The proportion of points with the same height among the different 3DSDM pairs was evaluated by means of its Hi, calculated for the Δh range between 0–0.5 mm with an interval of 0.05 mm.

**Figure 3:**
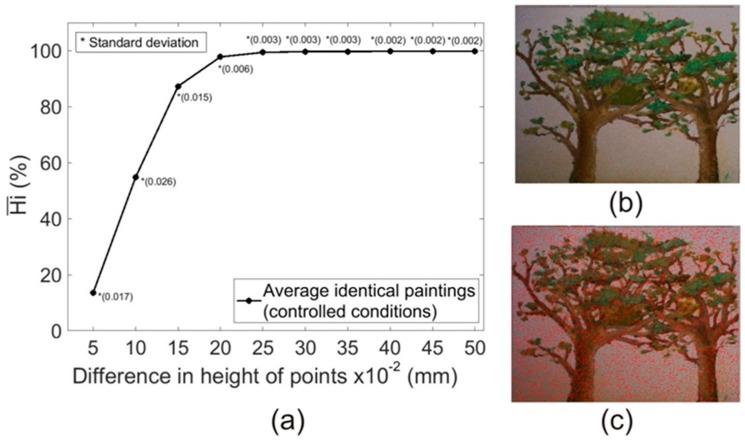
Experimental precision of the SL system analyzed with the same calibration conditions ($\bar{Hi}$) obtained for all the different 3DSDM pairs of identical samples for a Δh range of 0–0.5 mm with an interval of 0.05 mm (*n* = 12). (**b**) Example of a 3DSDM comparison (Sample C: C1–C2 pair) in which the red dots indicate a Δh in both longer than 0.25 mm. (**c**) Same 3DSDM comparison in which the red dots indicate a Δh in both longer than 0.15 mm.

Figure 3a shows the arithmetic mean of the Hi values ($\bar{Hi}$) obtained for each 3DSDM pair comparison of identical samples (*n* = 12) in the Δh range of 0–0.5 mm with an interval of 0.05 mm. One can see that, on average, 87.3% of the points of a given 3DSDM had a Δh < 0.15 mm with respect to another 3DSDM of the same sample. The same applies to 99.4% of the points for Δh < 0.25 mm and in 99.8% for Δh < 0.50 mm. According to these results, it can be considered reasonable to accept an experimental precision of 0.25 mm for the 3DSDM obtained with the calibration of the DAVID SLS-1 already mentioned, since the value of $\bar{Hi}$ was very close to 1 ($\bar{Hi}\;$= 0.994 ± 0.003; *n* = 12). Note that the precision estimated by us (0.25 mm) was similar to the optimum shown in the manufacturer’s technical specifications which is 0.1% of the object size (0.23 mm in our case). The low standard deviation shows the high consistency in the results obtained. Figure 3b,c show two representative examples of our 3DSDM pair comparison, specifically from sample C (C1 versus C2). The points that show different heights comparing both 3DSDMs are marked in red. It can be seen that many points show Δh > 0.15 mm (Figure 3c) among both 3DSDMs, but in the case of Δh > 0.25 mm, the number of points was residual (Figure 3b).

In the second step, we analyzed the potential of this SL system (i.e., DAVID SLS-1) to detect falsifications and to evaluate the deterioration of pictorial artworks. This analysis provides information regarding two aspects: First, the experimental repeatability of the results, comparing the 3DSDM pairs of identical samples obtained by changing the calibration of the equipment and, thus, simulating that the 3DSDMs were obtained on different dates (e.g., comparing the A1–A’ pair). This first analysis would confirm if SL is an appropriate technology to obtain a fingerprint of a pictorial artwork. Second, by comparing the 3DSDM of different pairs of samples; it could be verify if SL is capable of detecting small differences among apparently equal samples, ensuring that this technology can be used as a tool to detect falsifications. Especially important is the comparison between the 3DSDMs of the C1–D1 pair, where the differences in the relief between two equal pictorial artworks painted by the same artist would be detected. In this case, the comparison among all the samples was taken into account (i.e., 10): four corresponding to the sample with different calibration conditions (A1A’, B1B’, C1C’, and D1D’) and six corresponding to different samples with different calibration conditions, considering the first 3DSDMs of each sample (A1B1, A1C1, A1D1, B1C1, B1D1, and C1D1).

**Figure 4:**
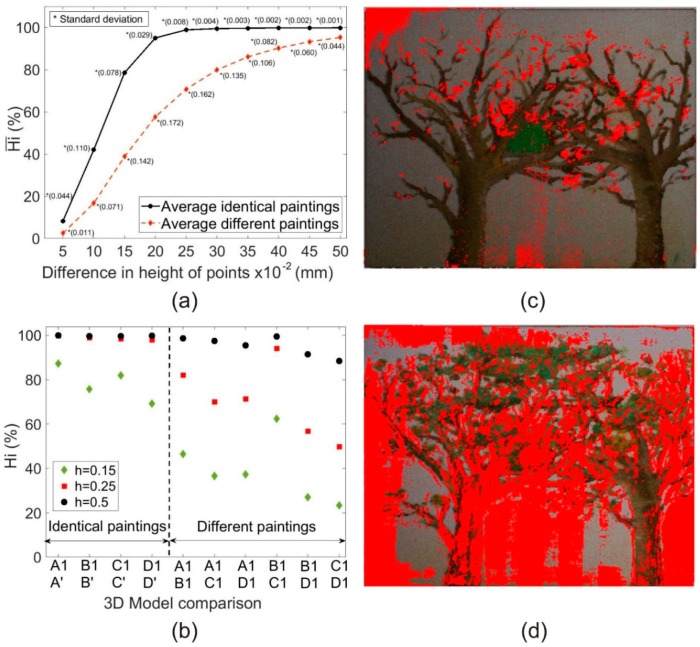
Results of the potential use of the SL system to detect falsifications or deteriorations of pictorial artworks: (**a**) $\bar{Hi}$ for all the different 3DSDM comparisons of identical (4 pairs, continuous black line) and different (6 pairs, discontinuous red line) samples for a Δh range of 0–0.5 mm, with an interval of 0.05 mm (*n* = 10). (**b**) 3DSDM comparison of identical (left) and different (right) samples, with indicators of their Hi for Δh = 0.15 mm (green diamond), Δh = 0.25 mm (red square), and Δh = 0.5 mm (black circle). (**c**) 3DSDM comparison example between samples B and C (i.e., B1C1 pair); red dots indicate a Δh in both 3DSDMs longer than 0.25 mm. (**d**) 3DSDM comparison example between samples C and D (i.e., C1D1 pair); red dots also indicate a Δh in both longer than 0.25 mm.

The results of the experimental repeatability can be seen in Figure 4a where $\bar{Hi}$ was evaluated for two cases: identical samples (*n* = 4, e.g., A1A’) and different samples (*n* = 6, e.g., A1B1). Again, the proportion of similar points among 3DSDMs of different samples was evaluated for a Δh range of 0–0.5 mm with an interval of 0.05 mm. One can see in Figure 4a that, on average, 78.6% of the points had a Δh < 0.15 mm among the two 3DSDMs of the same sample but with different calibration conditions. The same happens to 98.9% of the points for a Δh < 0.25 mm and in 99.9% for a Δh < 0.5 mm. However, these $\bar{Hi}$ values decreased in the case of the 3DSDM comparison of different samples: 38.8% of the points for a Δh < 0.15 mm, 70.8% of the points for a Δh < 0.25 mm, and in 95.3% for a Δh < 0.5 mm. The greatest difference between identical and different paintings was in the Δh range of 0.15–0.25 mm. However, it is necessary to point out that for Δh = 0.15 mm, many of the points were not identified as similar, so the sensitivity of the method would be overestimated.

On the left side of Figure 4b, we show the 3DSDM comparison for pairs of identical samples with different calibration conditions representing the Hi of each pair. One can see that this value is between 60–90% for a Δh = 0.15 mm which is not acceptable for detecting falsifications or deteriorations of pictorial artworks. However, for Δh = 0.25 mm, Hi values were close to the maximum possible value (Hi = 1) in all the comparisons carried out, providing a high precision for the SL system, even with different calibration conditions.

On the right side of Figure 4b the results of the Hi values for the 3DSDM comparison of different samples with the same calibration conditions are represented. Among all the results, we highlight B1C1. In this case, the Hi value that we accepted as the precision of the method (Δh = 0.25 mm) was very high. This was because the (small) differences between samples B and C were only the foliage of the trees, and, consequently, the similarity was high. This result can be associated to the study of the deterioration of a pictorial artwork: small differences in the 3DSDMs of the same sample obtained on different dates could mean the deterioration of some parts of the sample. The results of this 3DSDM comparison can be seen in Figure 4c where Δh > 0.25 mm is represented in red color. Some of the red sections are due to the existence of leaves in sample C while others indicate deteriorated areas of the painting (upper left corner and lower central part). On the other hand, while at first glance C1D1 may look similar, the analysis shows that their 3DSDMs are completely different, since Δh < 0.25 mm is really low (approximately 48%). This result allows us to affirm with certainty that sample D is a bad copy of sample C. The 3DSDM comparison for this case can be seen in Figure 4d where values for the Δh > 0.25 mm are distributed throughout the sample. Finally, note that in Figure 4a and due to the influence of different calibration conditions, $\bar{Hi}$ values for Δh = 0.25 mm decreased slightly when compared to that obtained with the same calibration conditions ($\bar{Hi}$ = 0.994 ± 0.003; *n* = 12), although it is confirmed best to distinguish between identical ($\bar{Hi}$ = 0.989 ± 0.008; *n* = 4) and different ($\bar{Hi}$ = 0.708 ± 0.162; *n* = 6) samples. Other Δh were not that useful for distinguishing between identical and different samples, as Δh values shorter than 0.25 mm neglect many similar 3DSDM points of identical samples, while Δh values longer than 0.25 mm obtained more similar $\bar{Hi}$ between both groups (i.e., identical and different).

The limitations of this work include the relatively low dataset and the lack of previous reference studies of structured light systems with the aim of detection of falsifications in pictorial artworks. Because of that, the results obtained are preliminary and need to be completed with other studies that include more samples and pictorial techniques. Also, a comparison of the results obtained with the low-cost equipment used in this work with more sophisticated 3D modeling equipment, such as a high-precision triangulation laser scanner, will be considered in future studies.

## 5. Conclusions

This paper analyzed the minimum differences in height that can be distinguished with the SL system DAVID SLS-1. Structured light allows for the obtention of three-dimensional surface digital models (3DSDM) which provide geometrical information about the surface of the pictorial artworks with high precision in an easy and economical way. This information, together with preventive conservation measures against damage caused by external agents, should constitute the measures to ensure the maintenance of the artworks over the years.

The surface information obtained with this NDT represent a fingerprint of the painting and can be used to prevent deterioration or to authenticate pictorial artworks. Obviously, determining the precision of the SL system in obtaining digital models seems essential in its use for protection purposes. In this paper, we used the low-cost system DAVID SLS-1 to experimentally estimate its precision in oil paintings on canvas. This precision, defined as the difference in height between 3DSDM pair points, was set by us as 0.25 mm. In other words, for differences greater than 0.25 mm, DAVID SLS-1 can be used to detect deteriorations or falsifications in this kind of pictorial artworks. Finally, we presented an experimental example of the use of this system for authentication and conservation purposes, with good results. The methodology developed in this work can serve as a guide to determine the precision of any SL system with others more expensive and sophisticated including new versions of DAVID-SLS.
